# Cepharanthine Prevents Estrogen Deficiency-Induced Bone Loss by Inhibiting Bone Resorption

**DOI:** 10.3389/fphar.2018.00210

**Published:** 2018-03-27

**Authors:** Chen-he Zhou, Jia-hong Meng, Yu-te Yang, Bin Hu, Jian-qiao Hong, Zheng-tao Lv, Kun Chen, Boon Chin Heng, Guang-yao Jiang, Jian Zhu, Zhao-hui Cheng, Wei Zhang, Le Cao, Wei Wang, Wei-liang Shen, Shi-gui Yan, Hao-bo Wu

**Affiliations:** ^1^Department of Orthopedic Surgery, Second Affiliated Hospital, School of Medicine, Zhejiang University, Hangzhou, China; ^2^Orthopedic Research Institute of Zhejiang University, Hangzhou, China; ^3^Department of Orthopedics, Tongji Hospital, Tongji Medical College, Huazhong University of Science and Technology, Wuhan, China; ^4^Faculty of Dentistry, The University of Hong Kong, Pokfulam, Hong Kong, Hong Kong; ^5^Department of Orthopaedic Surgery, Taizhou First People's Hospital, Taizhou, China

**Keywords:** cepharanthine, osteoclasts, osteoporosis, Akt, JNK

## Abstract

Osteoporosis is a common health problem worldwide caused by an imbalance of bone formation vs. bone resorption. However, current therapeutic approaches aimed at enhancing bone formation or suppressing bone resorption still have some limitations. In this study, we demonstrated for the first time that cepharanthine (CEP, derived from *Stephania cepharantha* Hayata) exerted a protective effect on estrogen deficiency-induced bone loss. This protective effect was confirmed to be achieved through inhibition of bone resorption *in vivo*, rather than through enhancement of bone formation *in vivo*. Furthermore, the *in vitro* study revealed that CEP attenuated receptor activator of nuclear factor κB ligand (RANKL)-induced osteoclast formation, and suppressed bone resorption by impairing the c-Jun N-terminal kinase (JNK) and phosphatidylinositol 3-kinase (PI3K)-AKT signaling pathways. The inhibitory effect of CEP could be partly reversed by treatment with anisomycin (a JNK and p38 agonist) and/or SC79 (an AKT agonist) *in vitro*. Our results thus indicated that CEP could prevent estrogen deficiency-induced bone loss by inhibiting osteoclastogenesis. Hence, CEP might be a novel therapeutic agent for anti-osteoporosis therapy.

## Introduction

Constant bone remodeling involves a fine balance between osteoclast-mediated bone resorption vs. new bone formation by osteoblasts (Kawai et al., 2011). Osteoporosis, which is caused by an imbalance of osteoblasts and osteoclasts, is a common disease worldwide with high morbidity, particularly among postmenopausal women and old people, and is characterized by decreased bone mass, abnormal bone architecture, and an increased risk of fragility fracture (Ivaska et al., 2010). Currently, there are about 10 million patients in the USA and 27.6 million patients in Europe whom are afflicted with osteoporosis (Saito et al., 2017). The annual cost for the treatment of osteoporosis-related fragility fractures is estimated to increase up to 25 billion dollars by 2025 in the USA alone, which represents a substantial economic burden (Burge et al., 2007; Budhia et al., 2012).

The aims of current anti-osteoporotic therapies are to improve bone mineral density (BMD) and reduce the risks of fragility fracture (Cranney et al., 2002). The various anti-osteoporotic drug approved by the US Food and Drug Administration (FDA) includes anabolic drugs and anti-resorptive drugs, such as bisphosphonates, selective estrogen receptor modulators (SERMs), calcitonin, parathyroid hormone [1–34] (Teriparatide, PTH[1–34]), and monoclonal antibodies against the receptor activator of nuclear factor κB ligand (RANKL) (Saito et al., 2017). However, the adverse side-effects of the currently available anti-osteoporotic drugs are obvious. One of the major concerns of current anti-resorptive drugs are that these drugs not only reduce bone resorption efficiently, but also profoundly suppress bone formation, resulting in deceased bone remodeling and increased clinical complications, such as osteonecrosis of the jaw and other atypical fractures (Khan et al., 2009; Koh et al., 2010; Lotinun et al., 2013). Teriparatide is an anabolic drug which enhances bone formation to a greater extent than bone resorption. Nevertheless, teriparatide should not be administered for more than 24 months as recommended by the FDA; meanwhile, the application of teriparatide increases cost-effectiveness (Pfister et al., 2006; Omiya et al., 2017). It is imperative to find a cost-effective drug which can inhibit bone resorption effectively, whilst maintaining or even enhancing bone formation for the treatment of osteoporosis.

Cepharanthine (CEP) (Figure S1A), a natural product isolated from *Stephania cepharantha* Hayata, has been used in the clinic for the treatment of various acute and chronic conditions, such as radiation-induced leukopenia, alopecia areata, and thrombocytopenic purpura associated with multiple myeloma (Ohta and Morita, 1990; Morita et al., 2002; Rogosnitzky and Danks, 2011; Tabata et al., 2012). Moreover, CEP also exhibits other diverse pharmacological activities, including anti-inflammatory, anti-viral and anti-allergic effects (Okamoto et al., 2001; Furusawa and Wu, 2007; Zhou et al., 2012; Paudel et al., 2016). However, no serious adverse side-effects of CEP treatment have been reported to date, even in the tumor therapy, whereby higher doses of CEP have been used (Takahashi-Makise et al., 2009). Up to now, there is as yet no reported study on the effects of CEP on bone remodeling. In addition, CEP has demonstrated some effects on inhibiting Na^+^, K^+^-ATPase activity (Satoh et al., 2003). Since the Na^+^, K^+^-ATPase plays a crucial role in the mechanism of bone resorption (Baron et al., 1986), we hypothesize that CEP might inhibit osteoclast functions and subsequently affect bone remodeling. Here, we aimed to explore the effects of CEP on ovariectomy-induced osteoporosis and elucidate the underlying mechanisms involved.

## Results

### CEP prevented estrogen-deficiency induced bone loss *in vivo*

**Figure 1:**
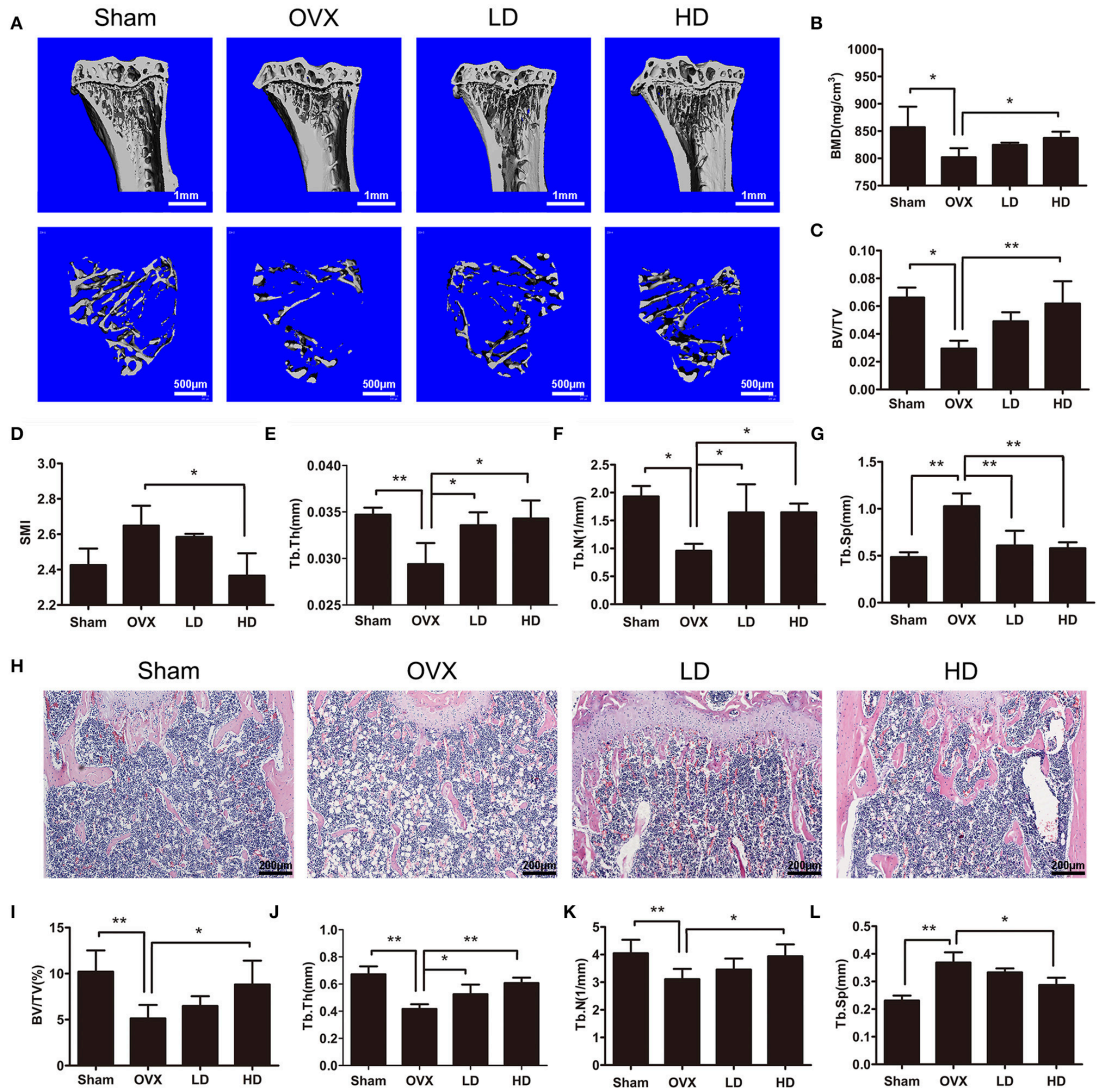
CEP prevented OVX-induced bone loss *in vivo*. **(A)** Representative 3D micro-CT images of trabecular bone of proximal tibias from each group were reconstructed. Scale bar = 1 mm and 500 μm **(B–G)** The BV/TV, Conn.D, SMI, Tb.N, Tb.Th, and Tb.Sp values of micro-CT data from each sample were obtained and analyzed **(H)**. H&E staining was performed for the histomorphological analysis. Scale bar = 200 μm **(I–L)** The BV/TV, Tb.N, Tb.Th, and Tb.Sp values of histomorphological sections were measured and quantified. LD and HD represent the dose of 5 and 20 mg/kg CEP, respectively. Values are expressed as mean ± *SD, n* = 10; ^*^P < 0.05, ^**^P < 0.01 vs. the control group.

To investigate the effects of CEP on bone remodeling, we established a murine model of ovarectomy (OVX)-induced osteoporosis and treated mice with either vehicle or different concentrations of CEP. The observed reduction of uterine weight and increase in body weight of OVX mice thus indicated that this model was successfully established (Figures S1B,C). Our micro-CT results of proximal tibia showed that the OVX procedure led to significant bone loss in mice with vehicle treatment (Figures 1A–G). However, a preventive effect against OVX-induced bone loss was observed in the groups treated with CEP, particularly in the high dose group (20 mg/kg) (Figure 1A). Bone mineral density (BMD) and bone volume/total volume (BV/TV) were slightly reversed in mice with low dose CEP treatment (5 mg/kg), although there were observed to be no significant differences upon analysis (Figures 1B,C). Moreover, the BMD, BV/TV, trabecular number (Tb.N), and trabecular thickness (Tb.Th) in the high dose treatment group were significantly higher than that of the OVX group, whereas the structural model index (SMI) and trabecular separation (Tb.Sp) were deceased (Figures 1B–G).

To further confirm the protective effects of CEP on OVX-induced osteoporosis, distal femurs were decalcified and evaluated using hematoxylin and eosin staining (H&E staining). As shown in Figure 1H, a smaller and thinner trabecular bone was observed after OVX surgery, whereas the administration of low dose and high dose of CEP mitigated the bone loss *in vivo*. The histomorphological quantification demonstrated that, there was a significant reduction in BV/TV, Tb.Th, and Tb.N, and an elevation in Tb.Sp in the OVX group (Figures 1I–L), as compared to the sham group. In accordance with the micro-CT results, histomorphological data revealed that CEP mitigated estrogen deficiency induced bone loss in a dose-dependent manner (Figures 1I–L). Collectively, these data exerted that CEP have a protective effect on estrogen-deficiency induced bone loss *in vivo*.

### CEP inhibited bone loss by suppressing osteoclastogenesis, and not by enhancing osteogenesis

**Figure 2:**
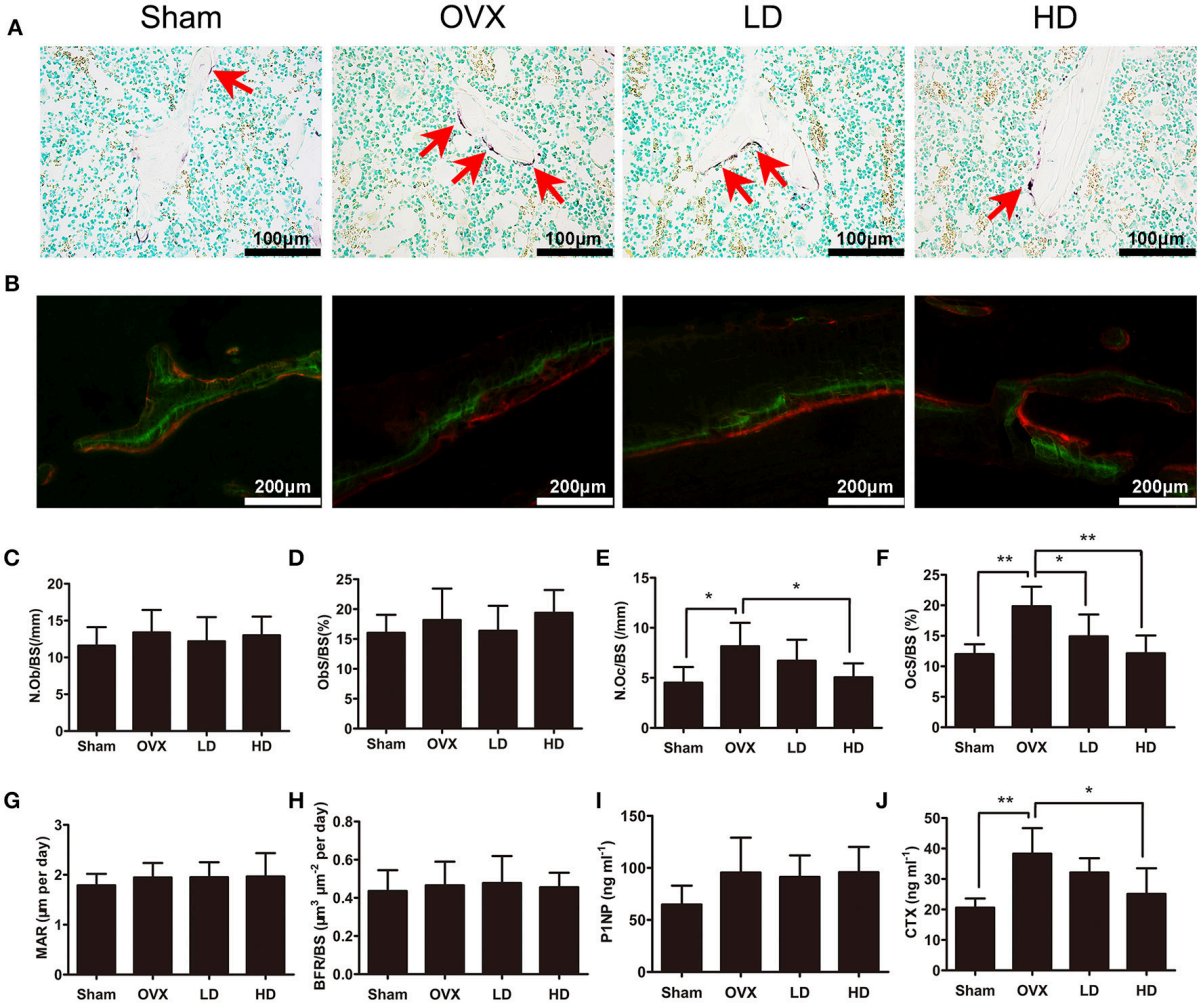
CEP impaired OVX-induced osteoclastogenesis *in vivo*, but does not affecting osteogenesis. **(A)** TRAP staining was performed on decalcified sections of distal femurs. Representative images of osteoclasts (red arrows) were measured. Scale bar = 100 μm **(B)** Representative images of calcein (green) and andalizarin red (red) labels were visualized. Scale bar = 200 μm **(C,D)** The N.Ob/BS and ObS/BS were measured with Toluidine Blue-stained sections. **(E,F)** The N.Oc/BS and OcS/BS were measured with TRAP-stained sections. **(G,H)** The MAR and BFR/BS were measured with undecalcified sections. **(I,J)** The serum levels of P1NP and CTX-1 were analyzed with ELISA. Values are expressed as mean ± *SD, n* = 10; ^*^P < 0.05, ^**^P < 0.01 vs. the control group.

Since osteoporosis is caused by an imbalance of bone formation vs. resorption during bone remodeling, the targets of anti-osteoporotic therapies are mostly focusing on either the inhibition of osteoclasts or the enhancement of osteoblast activities (Harslof and Langdahl, 2016). To determine how CEP mitigated estrogen deficiency-induced bone loss, we subsequently assessed the bone formation and bone resorption *in vivo* after CEP treatment. Our results showed that osteoclast formation was elevated significantly following OVX surgery (Figures 2A,E,F), whereas no significant differences were observed with osteoblast activity and bone formation in the OVX group, as compared to the sham group (Figures 2B–D,G,H). In the CEP treated groups, enhanced osteoclast formation following OVX was diminished in a concentration-dependent manner (Figures 2A,E,F). However, no significant differences in osteoblast number and activities, as well as bone formation were detected between the OVX and CEP-treated groups (Figures 2B–D,G,H). Serum markers for bone turnover, including type I collagen cross-linked C-terminal telopeptide (CTX-1) and procollagen 1 N-terminal peptide (P1NP), were measured as well. The results indicated that the levels of CTX-1, a marker for bone resorption, were markedly decreased in the high dose CEP treated group (Figure 2J). In contrast, CEP treatment did not result in any changes to the levels of P1NP, which is a marker for bone formation (Figure 2I). Taken together, our data thus indicated that CEP reversed estrogen deficiency induced osteoporosis by inhibiting osteoclastic bone resorption without enhancing bone formation.

### CEP impaired osteoclast formation, particularly at the early stage of differentiation

**Figure 3:**
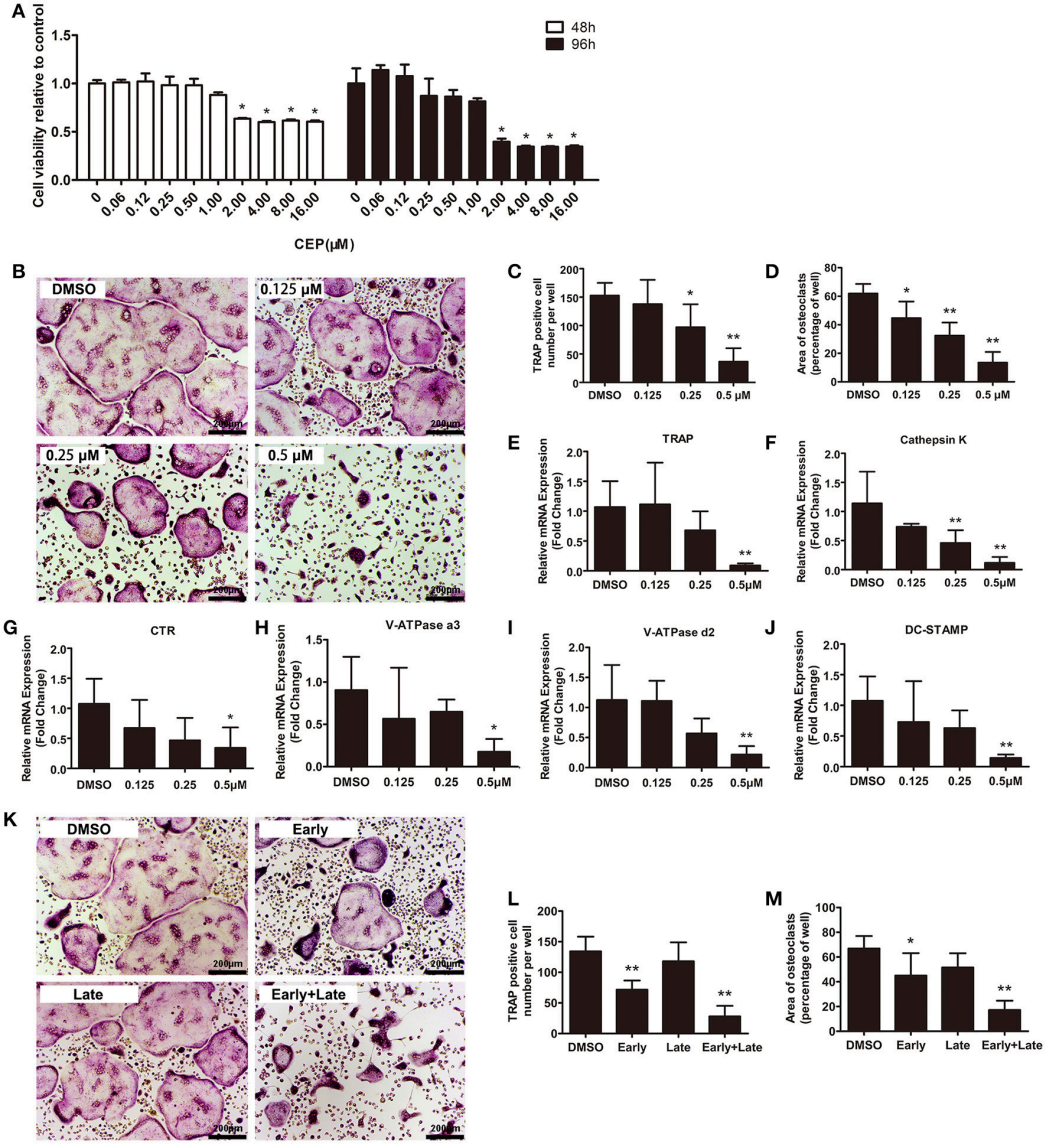
CEP attenuated RANKL-induced osteoclast formation *in vitro*. **(A)** BMMs were seeded at a density of 2 × 10^4^ cells/well and treated with the indicated concentrations of CEP in the presence of 30 ng/mL M-CSF for 48 or 96 h. The cell viability was quantified using the CCK8 assay. **(B)** TRAP staining was performed on BMMs treated with different doses of CEP in osteoclastogenic medium for 4 days. Scale bar = 200 μm. **(C,D)** The number and area of TRAP positive osteoclasts were analyzed. **(E–J)** The mRNA expression levels of Cathepsin K, TRAP, CTR, V-ATPase a3, V-ATPase d2, and DC-STAMP in BMMs treated with the indicated concentrations of CEP for 4 days were quantified. K BMMs were treated with 0.5 μM CEP for day 0–day 2 (Early-stage), day 2–day 4 (Late-stage), or day 0–day 4 (Early + Late stage) in osteoclastogenic medium. Scale bar = 200 μm. **(L,M)** The number and the area of osteoclasts were measured. All experiments were repeated independently for three times. Values are expressed as mean ± *SD*; ^*^P < 0.05, ^**^P < 0.01 vs. the control group.

To examine the effects of CEP on osteoclastogenesis, we next induced primary bone marrow-derived macrophages (BMMs) to differentiate into osteoclasts *in vitro*. Firstly, we evaluated the cytotoxicity of CEP by exposing the cells to varying concentrations of the drug (ranging from 0 to 16.00 μM) for 48 and 96 h. No toxicity of CEP was detected at lower than a concentration of 1 μM after 48 and 96 h treatment (Figure 3A). Next, we selected following the concentrations of CEP (0, 0.125, 0.25, and 0.5 μM) to investigate its effects on osteoclast formation. Figure 3B showed that a large number of osteoclasts were formed in the presence of 30 ng/mL macrophage colony-stimulating factor (M-CSF) and 50 ng/mL RANKL, as evidenced by the presence of tartrate-resistant acid phosphatase (TRAP) positive multinucleated cells after 4 days of treatment (Figure 3B). However, the 4-day treatment with CEP resulted in a decreased number and smaller size of osteoclasts in a dose-dependent manner, which thus indicated that CEP inhibited osteoclast formation (Figures 3B–D). The expression levels of osteoclastic specific genes, including TRAP, Cathepsin K, Calcitonin receptor (CTR), V-ATPase a3, V-ATPase d2, and dendritic cell-specific transmembrane protein (DC-STAMP), were markedly suppressed by CEP treatment in a dose-dependent manner after 5-days of osteoclast differentiation (Figures 3E–J).

To determine at which particular stage CEP exerted its effects on the process of osteoclast formation, we induced BMMs in osteoclastogenic medium with either vehicle or 0.5 μM CEP from day 0 to day 2 (early stage), day 2 to day 4 (late stage), or day 0 to day 4 (early + late stage). As shown in Figures 3K–M, significant decreases of TRAP positive cell number and size were observed at the early-stage and the early + late stage CEP treatment groups. In contrast, no significant differences in TRAP positive cell number and size were detected in the late-stage CEP treatment group, as compared to the DMSO group. Hence, it can be deduced that CEP treatment suppressed osteoclast differentiation, particularly at the early stage of osteoclastogenesis.

### F-actin ring formation and bone resorption was suppressed by CEP

**Figure 4:**
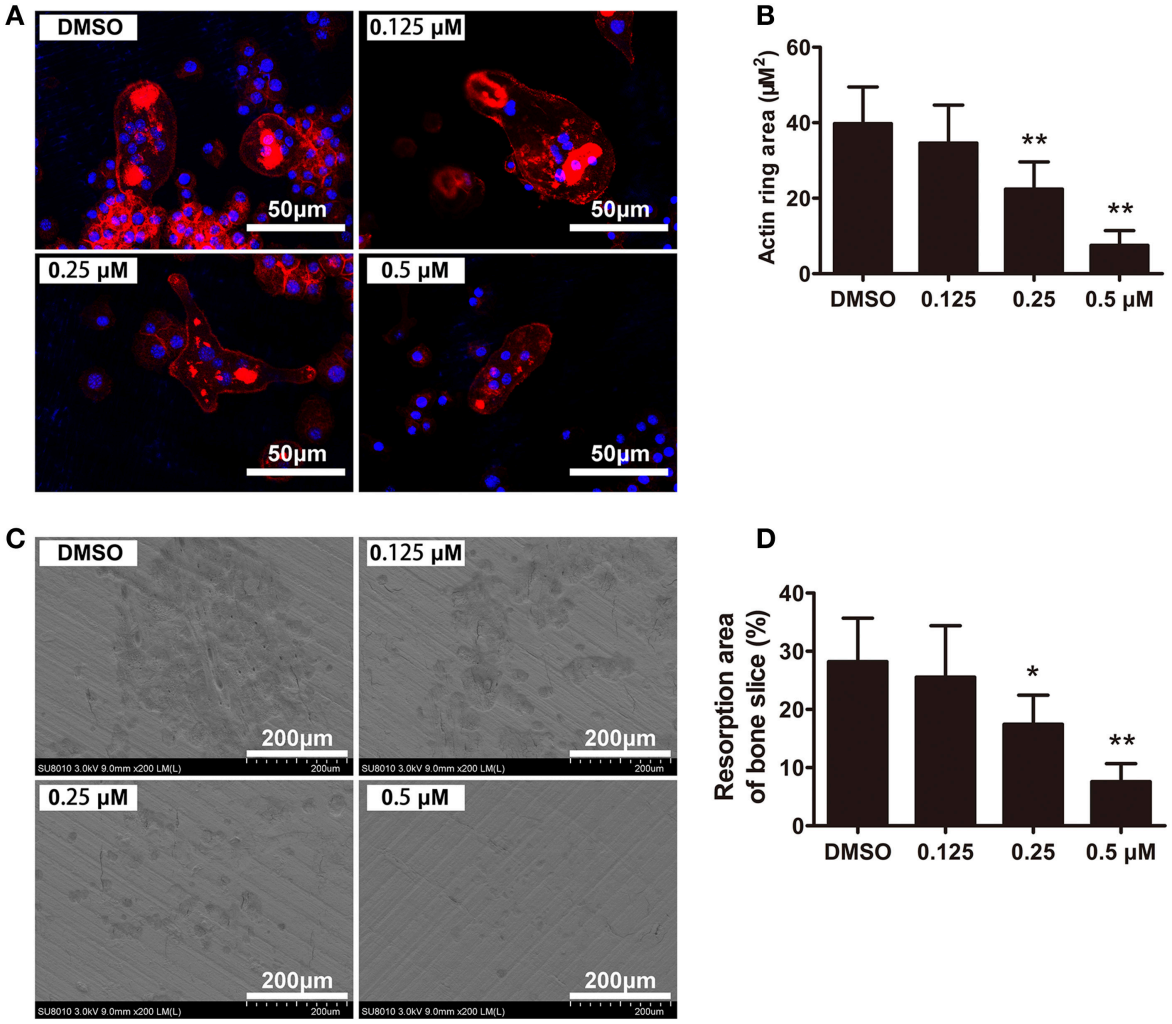
CEP inhibited F-actin ring formation and bone resorption *in vitro*. **(A)** BMMs were cultured with osteoclastogenic medium for 4 days and subsequently seeded on bone slices in the presence of indicated dilutions of CEP for another 48 h. Cells were fixed and stained to detect F-actin rings. Scale bar = 50 μm. **(B)** The area of the F-actin rings was quantified. **(C)** Cells were removed from bone slices and bone resorption pits were observed using SEM. Scale bar = 200 μm. **(D)** The area of bone resorption pits was measured. Values are expressed as mean ± *SD*; ^*^P < 0.05, ^**^P < 0.01 vs. the control group.

To further investigate the effects of CEP on osteoclast functions, we next evaluated the F-actin ring formation and performed bone resorption pit analyses. F-actin ring, which is the most characteristic and representative marker of resorption, reflects the functionally polarized status of osteoclasts (Ng et al., 2013). Characteristic osteoclastic F-actin rings were formed upon treatment with vehicle, whereas smaller and pleomorphic F-actin rings were observed in the CEP treatment group, in a dose-dependent manner (Figures 4A,B). After removing the cells, we next examined the resorption pits on the bone slices using scanning electron microscope (SEM). As shown in Figure 4C, extensive bone resorption pits were observed in the control group. In contrast, a dose-dependent reduction in the resorption area was observed upon treatment with varying concentrations of CEP (Figures 4C,D). Therefore, these data indicated that CEP suppressed bone resorptive activity of osteoclasts *in vitro*.

### CEP attenuated RANKL-induced activation of the JNK and AKT signaling pathways during osteoclastogenesis

**Figure 5:**
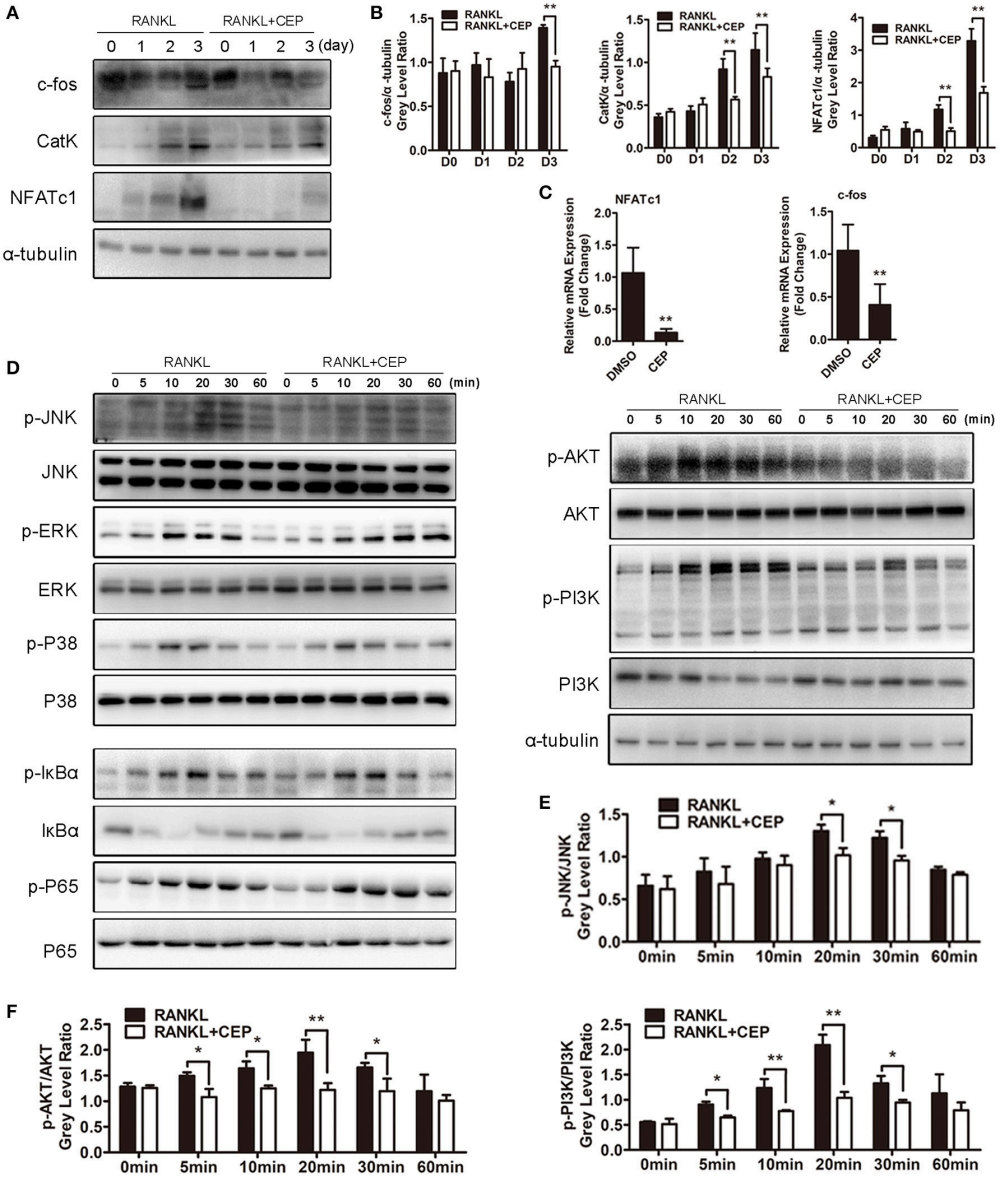
CEP suppressed RANKL-induced activation of the JNK and AKT pathways *in vitro*. **(A)** BMMs were cultured in osteoclastogenic medium with or without CEP treatment for 0, 1, 2, and 3 days. Cell lysates of samples from day 0 to day 3 were collected and analyzed by Western blotting using specific antibodies against NFATc1, c-Fos, and CatK. **(B)** The gray levels of NFATc1, c-Fos, and CatK were analyzed by normalization to α-tubulin. **(C)** After culture for 3 days, the cells were collected and the mRNA expression levels of NFATc1 and c-Fos were analyzed. **(D)** BMMs were pre-treated with or without CEP for 2 h and were subsequently treated with RANKL for 0, 5, 10, 20, 30, and 60 min. Cells were collected and lysates were analyzed by Western blotting using primary antibodies specific to p-p65, p65, p-ERK1/2, ERK, p-JNK1/2, JNK1/2, p-p38, p38, p-IκBα, IκBα, p-AKT, AKT, p-PI3K, PI3K, and α-tubulin. **(E,F)** The gray levels of p-JNK1/2, p-AKT, and p-PI3K were analyzed by normalization to total JNK1/2, AKT, and PI3K. Values are expressed as mean ± *SD*; ^*^P < 0.05, ^**^P < 0.01 vs. the control group.

To determine exactly which signaling pathways CEP inhibited, we further performed Western Blot and qPCR analyses. Nuclear factor of activated T cells c1 (NFATc1), which serves as the master transcriptional factor during osteoclast differentiation, is known to upregulate the expression of osteoclast-specific genes and proteins, depending on the mitogen-activated kinases (MAPK), TNF receptor-associated factor 6 (TRAF6)-NF-κB and c-Fos pathways (Takayanagi et al., 2002; Liu et al., 2016). Elevated protein expression levels of c-Fos, as well as its downstream targets NFATc1 and Cathepsin K, were observed after 1, 2, and 3 days of RANKL stimulation (Figures 5A,B). However, significant inhibition of c-Fos, NFATc1, and Cathepsin K expression were detected simultaneously in the CEP treatment group (Figures 5A,B). Analyses of the mRNA expression levels of NFATc1 and c-Fos on day 3 further confirm these results (Figure 5C).

Since the NF-κB, MAPK, and phosphatidylinositol 3-kinase (PI3K)-AKT signaling pathways play critical roles in osteoclast differentiation and activation (Boyle et al., 2003; Stevenson et al., 2011; Wu et al., 2017), we next explored the effects of CEP on RANKL-induced phosphorylation of these proteins in those pathways. As expected, upon treatment with RANKL, the phosphorylated levels of c-Jun N-terminal kinase 1/2 (JNK1/2), extracellular signal-regulated kinase 1/2 (ERK1/2), p38, factor of kappa light polypeptide gene enhancer in B-cells inhibitor alpha (IκBα), p65, AKT, and PI3K were all elevated and reached the maximal levels after 20 or 30 min of treatment, while the expression of IκBα was markedly reduced and reached the minimal levels after 10 min of treatment (Figures 5D,F, Figures S2A–E). Nevertheless, CEP treatment significantly inhibited RANKL induced phosphorylation of JNK, AKT, and PI3K (Figures 5D–F). No significant inhibition of RANKL-induced phosphorylation of ERK1/2, p38, IκBα, p65 were detected upon co-treatment with CEP, as compared to RANKL treatment alone (Figure 5D, Figures S2A–E).

A recent study indicated that nucleotide-binding domain and leucine-rich repeats C3 (NLRC3), a member of the NLR family, associated with PI3K, negatively regulate the activation of the PI3K-AKT signaling pathway (Karki et al., 2016). Due to the key role the PI3K-AKT signaling in osteoclastogenesis, we next explored the relationship between NLRC3 and osteoclast. As shown in Figures S3A,B, the mRNA and protein expressions of NLRC3 were reduced significantly during the process of osteoclast differentiation. However, CEP treatment inhibited the reduction of the expression of NLRC3 both at the early and late stages of osteoclastogenesis (Figures S3C,D).

Hence, these findings suggested that CEP could inhibit RANKL-induced activation of the JNK and PI3K-AKT signaling pathways, as well as the c-Fos and NFATc1 signaling cascades.

### The JNK and AKT agonists can partly reverse the suppressive effects of CEP on osteoclastogenesis

**Figure 6:**
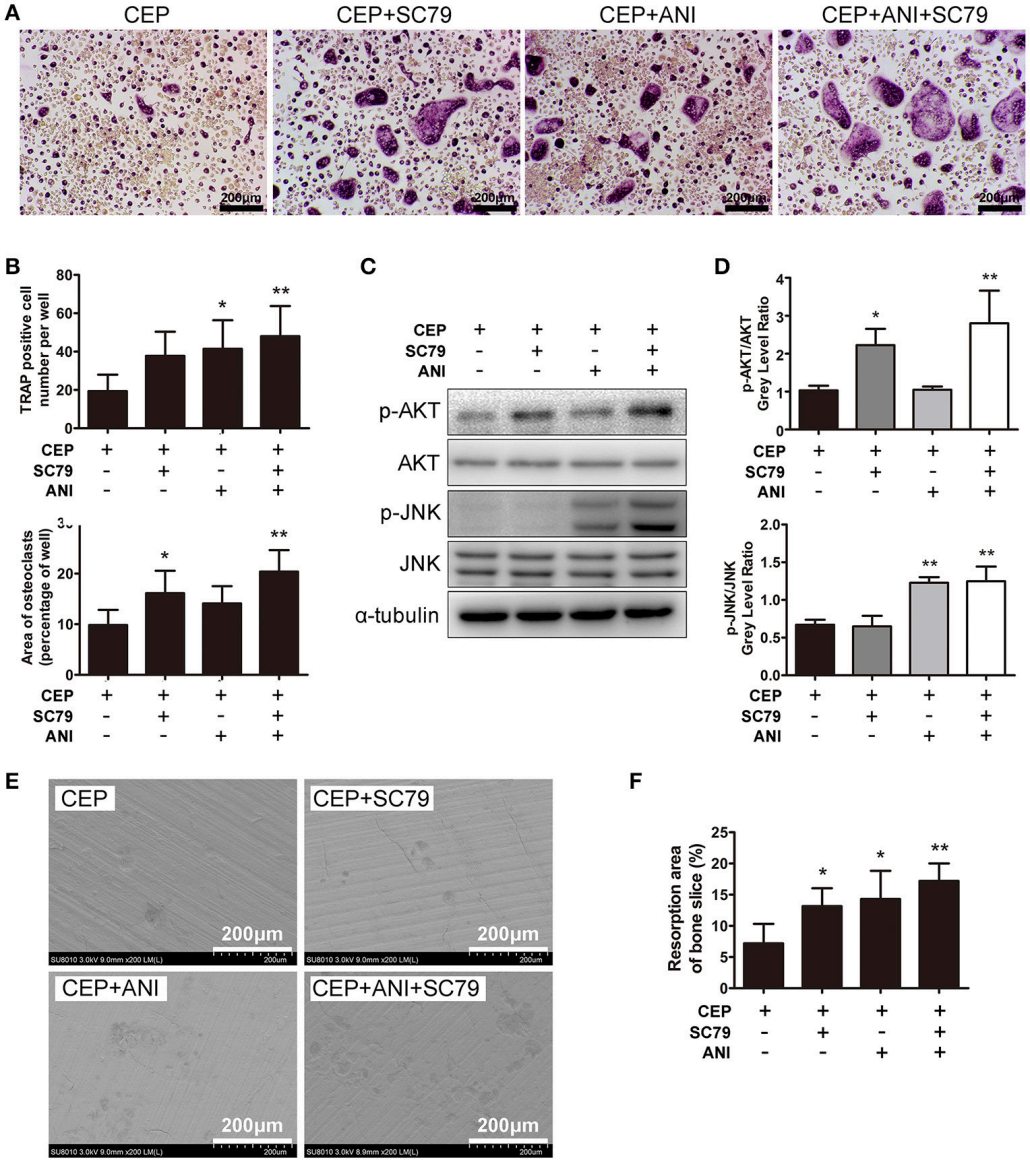
ANI and SC79 partly reversed the inhibitory effects of CEP on RANKL-induced osteoclastogenesis. **(A)** BMMs were cultured in osteoclastogenic medium containing CEP for 4 days in the presence of DMSO, ANI, and/or SC79. Cells were fixed and TRAP staining was performed. Scale bar = 200 μm. **(B)** The number and size of TRAP positive osteoclasts were quantified. **(C)** BMMs were pre-treated with CEP for 2 h and subsequently stimulated with RANKL in the presence of DMSO, ANI, and/or SC79 for 20 min. Cell lysates were collected and analyzed by Western blotting using primary antibodies specific to p-AKT, AKT, p-JNK1/2, and JNK. **(D)** The gray levels of p-JNK1/2 and p-AKT were analyzed by normalization to total JNK1/2 and AKT. **(E)** BMMs were cultured with osteoclastogenic medium for 4 days, and subsequently seeded on bone slices with CEP treatment in the presence of DMSO, ANI and/or SC79 for another 48 h. Bone resorption pits were observed under SEM. Scale bar = 200 μm **(F)** The area of the bone resorption pits was measured. Values are expressed as mean ± *SD*; ^*^P < 0.05, ^**^P < 0.01 vs. the control group.

As discussed earlier, CEP can strongly inhibite osteoclastogenesis *in vitro* through suppressing activation of the JNK and PI3K-AKT signaling pathways. To further validate these results, we investigated whether combining anisomycin (ANI, a JNK, and p38 agonist) and/or SC79 (an AKT agonist) together with CEP treatment can further rescue the inhibition of osteoclast differentiation. The TRAP staining results revealed that significant inhibition of osteoclast differentiation was detected in cells with CEP treatment alone (Figure 6A). However, in cells co-treated with ANI and/or SC79, an increased number and larger size of osteoclasts were observed, particularly in the group treated with CEP, ANI, and SC79 (Figures 6A,B). Also, the Western Blotting confirmed that the suppression of AKT and JNK phosphorylation by CEP was rescued in cells co-treated with ANI and/or SC79 (Figures 6C,D). We subsequently confirmed that co-treatment of SC79 and/or ANI could also reverse the inhibition of osteoclastic bone resorption by CEP and that the largest resorption pit area was detected in the SC79+ANI group (Figures 6E,F). Hence, it can be deduced that the inhibition of osteoclast formation and bone resorption following CEP treatment can be partially rescued by the JNK and AKT agonists.

## Discussion

The homeostasis of bone metabolism requires a fine balance of bone formation vs. resorption. During normal bone remodeling, osteoblasts generate new bone to completely refill the osteoclastic resorption lacunae (Lotinun et al., 2017). Hyperactive or hypoactive osteoclasts and/or osteoblasts induce abnormal bone remodeling, which results in serious bone disorders, such as osteopetrosis, osteoporosis, and osteogenesis imperfecta (Gatti et al., 2009; Charles and Aliprantis, 2014). Osteoporosis is the most common amongst these diseases, which afflicts millions of patients worldwide. Various drugs have been used clinically for the treatment of osteoporosis. However, the adverse side-effects limit the indications for the administration of these drugs (Khosla and Hofbauer, 2017). In this study, we demonstrated that CEP exerted the protective effects on estrogen deficiency induced osteoporosis by inhibiting osteoclast formation and bone resorption without enhancing or suppressing bone formation *in vivo*. Moreover, we further elucidated that CEP attenuated osteoclastogenesis by impairing the JNK and PI3K-AKT signaling pathways *in vitro*. Therefore, our data suggested that CEP might be a promising drug for osteoporosis treatment.

Current anti-osteoporotic drugs approved by FDA exhibit their protective effects on osteoporosis by promoting osteogenesis or suppressing osteoclastogenesis. As mentioned, the issues pertaining to suppressive effects of anti-resorptive drugs on osteogenesis and the enhancing effects of anabolic drugs om osteoclastogenesis still present major challenges in anti-osteoporotic therapy. Due to these current limitations, new therapeutic approaches should focus on more than bone resorption or bone formation alone. A number of natural compounds have exerted their potential on preventing osteoporosis (Chen et al., 2015; Xie et al., 2017). Nevertheless, the adverse effects of the long-term use of these compounds are unknown. CEP has been applied to clinical therapy for more than 40 years in Japan and no side-effects have been reported to date (Ita et al., 2008), further confirming its safety record with humans. In this study, an anti-osteoporotic effect was observed in our ovarectomized model upon treatment with CEP. As significantly increased trabecular bone volume, trabecular bone number, and reduced trabecular bone separation were detected by micro-CT and histomorphology in OVX mice after treatment with CEP, it was of interest to elucidate how CEP contributed to the suppression of estrogen deficiency-induced bone loss. As expected, CEP markedly inhibited OVX-induced increase in the number and size of osteoclasts *in vivo*, resulting in reduced bone resorption. However, there was neither enhancement nor reduction in the osteoblast number and bone formation rate. P1NP and CTX-1, the markers of bone formation and bone resorption, respectively, are commonly used to evaluate the efficacy of anti-osteoporotic treatments (Watts et al., 2008, 2010; Vasikaran et al., 2011). Our results further demonstrated that CEP inhibited OVX-induced elevation of serum CTX-1 levels, but no changes in serum P1NP levels were observed after treatment with CEP. These findings were in accordance with the histomorphological data described earlier. Therefore, the recovery of bone mass by CEP treatment in OVX mice was considered to be solely the result of inhibiting bone resorption.

As reported previously, CEP exerts its pharmacological action by inhibiting Na^+^, K^+^-ATPase activity (Satoh et al., 2003). Na^+^, K^+^-ATPase, which is highly expressed in osteoclasts, is considered to be associated with osteoclast-mediated bone resorption and is possibly coupled to secondary active calcium and/or proton transport proteins, such as the V-ATPase a3 subunit and V-ATPase d2 subunit (Baron et al., 1986). Additionally, Na^+^, K^+^-ATPase is also reported to play a role in facilitating the cell-cell fusion of osteoclast precursor cells in response to RANKL induction by upregulating DC-STAMP (Makihira et al., 2011). In our study, the results indicated that CEP, particularly at a dose between 0.125 and 0.5 μM, inhibited osteoclast formation potently in a dose-dependent manner, as well as the mRNA expression levels of proton transport proteins (V-ATPase a3 subunit and V-ATPase d2 subunit), DC-STAMP, TRAP, CTR, and Cathepsin K. Besides the suppression of osteoclast formation, CEP also exerts its therapeutic effects by impairing the bone resorptive capacity of mature osteoclasts.

Multiple signaling pathways are implicated in the process of osteoclastogenesis. JNK is a member of the MAPK family, being activated by RANKL-RANK signaling in osteoclasts or osteoclast precursors (Jimi et al., 1999). Upon binding to RANK, TRAF6 is trimerized and activates MAPKs and NF-κB signaling (Lee and Kim, 2003). JNK1, not JNK2, is then phosphorylated by its direct upstream kinase MAP kinase kinase 7 (MKK7), subsequently activating the transcription of activator protein-1(AP-1), which is essential for efficient osteoclastogenesis (Leibbrandt and Penninger, 2008). Our data showed that CEP significantly inhibited the phosphorylation of JNK, while no differences were observed in the phosphorylation of p38, ERK, and NF-κB. The activation of the AKT signaling pathway is achieved through the stimulation of both RANKL and M-CSF, which in turn regulates TRAF6-Src-PI3K interaction, ultimately modulating osteoclast survival and differentiation (Wong et al., 1999; Yuan et al., 2015). In our study, CEP markedly suppressed RANKL-induced phosphorylation of PI3K and AKT. To further confirm the inhibition of PI3K/AKT and JNK phosphorylation by CEP, we next utilized an AKT agonist (SC79) and a JNK agonist (ANI) to co-treat BMMs with CEP. As expected, the agonists partly reversed the suppressed phosphorylation of AKT and JNK, as well as inhibited osteoclast formation and reduced bone resorption. NLRC3 is a cytoplasmic sensor that regulates multiple biological functions and is known to be highly expressed in spleen and leukocyte in humans (Schneider et al., 2012; Karki et al., 2016; Tocker et al., 2017). It interacts with p85 subunits of PI3K, and blocks the interaction between PI3K p85 and p110α, following suppression of the activation of the PI3K-AKT signaling in cancer (Karki et al., 2016). As expected, NLRC3 expression was observed to be reduced during the process of osteoclast differentiation. However, CEP has been shown to reverse the decreased expression of NLRC3, which was similar to its effects in cancer. This might be due to the inhibition of the PI3K-AKT signaling in osteoclasts. Hence, it can be deduced that CEP impaired osteoclastogenesis through PI3K/AKT and JNK inhibition.

In conclusion, this study demonstrated that CEP exhibits its positive efficacy in preventing estrogen deficiency induced-bone loss by attenuating bone resorption without affecting bone formation *in vivo*. Moreover, we confirmed that CEP exerts its inhibitory effects on osteoclast formation and osteoclastic bone resorption by impairing the PI3K/AKT and JNK signaling pathways. Hence, we can conclude that CEP might be a novel and promising therapeutic agent for preventing osteoporosis.

## Materials and methods

### Media and reagents

CEP, ANI, and SC79, were purchased from Selleck Chemicals (Houston, USA), dissolved in DMSO and stored at −20°C, prior to being utilized in experiments. Alpha modification of Eagle's medium (α-MEM), FBS, and penicillin/streptomycin were purchased from Gibco-BRL (Sydney, Australia). The cell counting kit (CCK-8) was purchased from Dojindo Molecular Technology (Kumamoto, Japan). Kits for quantitative PCR experiment, including The Prime Script RT reagent kit and SYBR® Premix Ex Taq™ II, were purchased from TaKaRa Biotechnology (Otsu, Shiga, Japan). Recombinant murine soluble RANKL and M-CSF were obtained from R&D systems (Minneapolis, MN). Specific primary antibodies against ERK, JNK, p38, phosphorylated p-ERK, p-JNK, p-p38, IκBα, p-IκBα, p65, p-p65, PI3K, p-PI3K, AKT, p-AKT, and α-Tubulin were procured from Cell Signaling Technology (Cambridge, MA, USA); while primary antibodies specific for c-Fos, NFATc1, and Cathepsin K were purchased from Santa Cruz Biotechnology (Santa Cruz, CA, USA). Primary antibodies specific for NLRC3 were purchased from Abcam (Abcam, Cambridge, UK). HRP-conjugated secondary antibodies against rabbit IgG, and mouse IgG were purchased from Boster Biological Technology co. (Wuhan, China) and secondary antibody against goat IgG was procured from Santa Cruz Biotechnology (Santa Cruz, CA, USA). Bone slices, and commercial ELISA kits for assaying CTX-1 and P1NP were obtained from Immunodiagnostic Systems Limited (Boldon, UK). Rhodamine-conjugated Phalloidin, Diagnostic Acid Phosphatase kit for TRAP and all other reagents were purchased from Sigma-Aldrich (St. Louis, MO, USA), unless stated otherwise.

### Establishment of OVX-induced osteoporosis model

The animal experimental protocol was designed and performed in accordance with the Guide for the Care and Use of Laboratory Animals promulgated by the United States National Institutes of Health (NIH), and was approved by the Animal Care and Use Committee of Zhejiang University. Forty 8-week old female C57BL/6 mice were randomly divided into four groups: Sham (Sham operation with PBS injection), OVX (OVX surgery with PBS injection), low-dose CEP (OVX surgery with 5 mg/kg CEP injection), and high-dose CEP (OVX surgery with 20 mg/kg CEP injection). Mice were anesthetized with intramuscular xylazine (2 mg/kg) and ketamine (50 mg/kg) and either sham operation or OVX surgery was performed as described previously (Chen et al., 2015). All mice were injected with PBS/CEP twice a week and sacrificed with an overdose injection of pentobarbital (90 mg/kg, Sigma Chemical Co., St. Louis, MO, USA) after a total period of 6 weeks treatment. Calcein green (20 mg/kg; sigma) and alizarin red (30 mg/kg) were injected 9 and 2 days respectively before sacrifice. Bilateral tibias and left femurs were dissected and fixed in 4% (w/v) paraformaldehyde solution for histomorphology and micro-CT analysis. The murine blood was also collected and the isolated serum was stored at −80°C for ELISA analysis.

### Micro-CT scanning and analysis

All the left tibias were collected for micro-CT scanning at an isometric resolution of 5 μm with a Scanco μCT100 instrument (Scanco Medical, Bassersdorf, Switzerland). Three dimensional (3D) reconstructions of proximal tibias were performed with a multilevel threshold procedure (thresholds ranging from 220 to 520). The region at a distance of 0.5 mm below the growth plate was regarded as the region of interest (ROI) for quantitative analyses, including BMD, BV/TV, Conn.D, SMI, Tb.N, Tb.Th, and Tb.Sp.

### Histomorphological analysis

All the left femurs from mice were decalcified and embedded into paraffin as previously described (Zhou et al., 2017). Specimens were cut into 5 μm-thick sections along the coronal plane from anterior to posterior. Subsequently, H&E staining, Toluidine Blue, and TRAP staining were performed using light microscopy (Olympus BX51, Tokyo, Japan) for the evaluation of bone volume, osteoblasts, and osteoclasts, respectively. All the right tibias were embedded in modified methyl methacrylate without decalcification. Five micrometer thick sections were cut and the perimeters for bone formation were observed under fluorescence microscopy (Leica DM5 500B, Leica Microsystems, Bensheim, Germany) as previously reported (Liu et al., 2016). BV/TV, Tb.N, Tb.Th, Tb.Sp, the number of osteoblasts normalized to the bone surface (N.Ob/BS), the surface area of osteoblast to bone surface area (ObS/BS), the number of osteoclasts normalized to the bone surface (N.Oc/BS), the surface area of osteoclast to bone surface area (OcS/BS), the mineral apposition rate (MAR) and bone formation rate (BFR/BS) within ROI were quantified using Image J software.

### Bone turnover analysis

The blood was collected and centrifuged at 2,000 rpm for 20 min to isolate serum as previously described (Zhou et al., 2017). Subsequently, serum concentrations of CTX-1 and P1NP were measured according to the ELISA kit manufacturer's protocol. All samples were analyzed three times.

### BMMs isolation, cell viability assay, and osteoclast differentiation *in vitro*

Primary bone marrow cells were extracted from the long bones of male 6 week old C57BL/6 mice and cultured in α-MEM containing 10% (v/v) FBS, 1% (v/v) penicillin/streptomycin (complete α-MEM), and 30 ng/mL M-CSF for 2 days in an incubator at 37°C with 5% CO_2_.

To assay the cytotoxicity of CEP, a cell count of 2 × 10^4^ cells/well were seeded and cultured in a 96-well plate for 24 h. Subsequently, cells were then cultured in complete α-MEM supplemented with varying doses of CEP (0, 0.06, 0.12, 0.25, 0.50, 1.00, 2.00, 4.00, 8.00, and 16.00 μM) for 48 or 96 h. Then, the CCK-8 assay was performed by adding 10 μL of CCK-8 buffer per well, and the plate was incubated for another 2 h. The optical density (OD) was analyzed at a wavelength of 450 nm (650 nm reference) using an ELX800 absorbance microplate reader (Bio-Tek Instr., Winooski, VT, USA).

BMMs, seeded at a density of 8 × 10^3^ cells/well in a 96-well plate, were cultured in complete α-MEM containing 30 ng/mL M-CSF and 50 ng/mL RANKL (osteoclastogenic medium) in the presence of indicated dilutions of CEP with or without 2.5 ng/mL ANI (a JNK agonist) and/or 8 μg/mL SC79 (a AKT agonist). The osteoclastogenic medium was changed every 2 days for the induction of osteoclastogenesis. After 4 days of induction culture, the cells were fixed with 4% (w/v) PFA and stained with TRAP staining kit according to the manufacturer's introduction. Multinuclear cells with more than five nuclei were counted as mature osteoclasts under light microscopy and the cell size was measured as well.

### F-actin ring formation assay and bone resorption assay

BMMs were cultured in osteoclastogenic medium for 4 days until mature osteoclasts were formed. BMMs-derived osteoclasts were digested and seeded uniformly on bovine bone slices overnight as previously described (Ng et al., 2013). Subsequently, cells were cultured with osteoclastogenic medium in the presence of indicated dosages of CEP with or without 2.5 ng/mL ANI (a JNK agonist) and/or 8 μg/mL SC79 (a AKT agonist). After 48 h, cells were fixed in 4% (w/v) PFA and stained with rhodamine-conjugated phalloidin and DAPI. F-actin rings were observed and measured using a NIKON A1Si spectral detector confocal system. Subsequently, cells were then removed using a soft brush and resorption pits were visualized using a scanning electron microscope (SEM). The size of F-actin rings and resorption pits were quantified using the ImageJ software.

### RNA isolation and qPCR

BMMs were cultured in a 6-well plate at a density of 10 × 10^4^ cells/well to induce differentiation to osteoclasts in the presence of indicated doses of CEP for 4 days. The extraction of total RNA was performed using an RNAeasy Mini Kit (Qiagen, Valencia, CA, USA) according to the manufacturer's instructions. cDNA was synthesized and qPCR was performed as described previously (Zhou et al., 2017). GAPDH was utilized as the housekeeping gene and all experiments were repeated independently at least three times. The primer sequences were as follows: GAPDH, forward 5′-ACCCAGAAGACTGTGGATGG-3′ and reverse 5′-CACATTGGGTAGGAACAC-3′; Cathepsin K, forward 5′-CTTCCAATACGTGCAGCAGA-3′ and reverse 5′-TCTTCAGGGCTTTCTCGTTC-3′; CTR, forward 5′-TGCAGACAACTCTTGGTTGG-3′ and reverse 5′-TCGGTTTCTTCTCCTCTGGA-3′; TRAP, forward 5′-CTGGAGTGCACGATGCCAGCGACA-3′ and reverse 5′-TCCGTGCTCGGCGATGGACCAGA-3′; c-Fos, forward 5′-CCAGTCAAGAGCATCAGCAA-3′ and reverse 5′-AAGTAGTGCAGCCCGGAGTA-3′; NFATc1, forward 5′-CCGTTGCTTCCAGAAAATAACA-3′ and reverse 5′-TGTGGGATGTGAACTCGGAA-30′; V-ATPase d2, forward 5′-AAGCCTTTGTTTGACGCTGT-3′ and reverse 5′-TTCGATGCCTCTGTGAGATG-3′; V-ATPase a3, forward 5′-TGGCTACCGTTCCTATCCTG-3′ and reverse 5′-CTTGTCCGTGTCCTCATCCT-3′; DC-STAMP, forward 5′-AAAACCCTTGGGCTGTTCTT-3′ and reverse 5′-AATCATGGACGACTCCTTGG-3′. NLRC3 forward 5′-GTCAGCTGCTACAAGTCCGGGAC-3′ and reverse 5′-GAGCCTCAGAGTGCTTCGGTATCC-3′.

### Western blot analysis

Primary BMMs, constituted in complete α-MEM medium with 30 ng/mL M-CSF, were seeded into 6-well plates at a density of 100 × 10^4^ cells/well, and cultured for 24 h. After being pre-treated with DMSO or 0.5 μM CEP for 2 h, BMMs were subsequently stimulated with 50 ng/mL RANKL for 0, 5, 10, 20, 30, or 60 min, to elucidate the exact signaling pathways that CEP affected. For the rescue assay, the BMMs were seeded at the same density and pre-treated with 0.5 μM CEP, together with DMSO, 2.5 ng/mL ANI and/or 8 μg/mL SC79 for 2 h, followed by stimulation with RANKL for another 20 min. To investigate the effects of CEP treatment on the protein expression levels of NFATc1, c-Fos, and Cathepsin K, the BMMs were seeded in 6-well plates at a density of 10 × 10^4^ cells/well in osteoclastogenic medium with DMSO or 0.5 μM CEP for 0, 1, 2, and 3 days. The cells were then collected and the total protein was extracted using radio immune precipitation assay (RIPA) lysis buffer (Sigma Aldrich, St Louis, MO, USA). Subsequently, the supernatants were collected and the concentrations of protein were measured with a bicinchoninic acid assay kit (BCA, Thermo Fisher, MA, USA). Ten micrograms of total protein of each sample was separated by SDS-PAGE and then transferred to polyvinylidene difluoride membranes (Millipore, Bedford, MA, USA). Specific primary antibodies were used to probe these membranes with overnight incubation at 4°C followed by 1 h non-specific blocking with 5% (w/v) skimmed milk in Tris-buffered saline-Tween (Invitrogen, San Diego, CA, USA). After two washes, the membranes were incubated with the appropriate secondary antibodies at 4°C for 2 h. Subsequently, specific bands were detected by a Bio-Rad XRS chemiluminescence detection system (Bio-Rad, Hercules, CA, USA) using electrochemical luminescence reagent (ECL) (Millipore, Billerica, MA, USA) on the membranes. The quantification of the bands was performed using the ImageJ software.

### Statistical analysis

All data were analyzed by two investigators blind to details of the treatment. Results were expressed as mean ± *SD*. All experiments were independently repeated at least three times and the results were analyzed using SPSS 16.0 software (SPSS, Chicago, IL, USA). One-way ANOVA with post hoc Newman–Keuls test was performed to analyze differences in multiple group comparisons while an unpaired *t*-test was performed for the comparisons between two groups. The threshold of statistical significance was set at ^*^p < 0.05 and ^**^p < 0.01.

## Author contributions

CZ, HW, SY, and WS: designed the experiment; CZ, JM, YY, JH, GJ, and WZ: performed the experiment; BH, ZL, KC, JZ, and LC performed the measurement and analysis; CZ, ZC, WW, WS, and HW: drafted the manuscript; BCH: revised the manuscript.

### Conflict of interest statement

The authors declare that the research was conducted in the absence of any commercial or financial relationships that could be construed as a potential conflict of interest.
