# Supplementary material for: Cepharanthine Prevents Estrogen Deficiency-Induced Bone Loss by Inhibiting Bone Resorption
Source: Front Pharmacol. 2018 Mar 27;9:210. doi: 10.3389/fphar.2018.00210 (PMC5880888; doi:10.3389/fphar.2018.00210)
Supplement: Supplementary file 1 [file Presentation1.pdf]

Cepharanthine prevents estrogen deficiency-induced bone loss by inhibiting bone resorption

Chen-he Zhou[1,2,3¶], Jia-hong Meng[1,2¶], Yu-te Yang[1,2¶], Bin Hu[1,2], Jian-qiao Hong[1,2], Zheng-tao Lv[3,4], Kun Chen[3,4], Boon Chin Heng[5], Guang-yao Jiang[1,2], Jian Zhu[1,2], Zhao-hui Cheng[6], Wei Zhang[1,2], Le Cao[1,2], Wei Wang[1,2], Wei-liang Shen[1,2]**, Shi-gui Yan[1,2]**, Hao-bo Wu[1,2]*

Figure S1

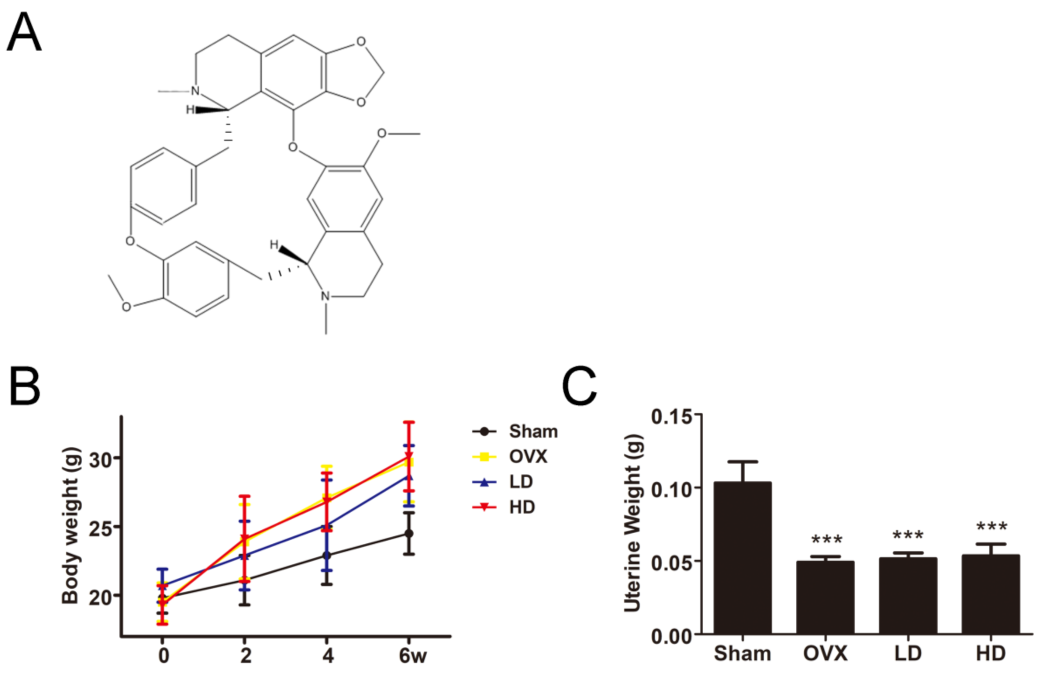

OVX induced increased body weight and reducteduterus weight. (A) The chemical structure of CEP. (B) The body weight of mice 0-6 weeks after OVX in Sham, OVX, Low dose and High dose groups. (C) The weight of the mouse uteri at the time of sacrifice in all four groups. Values are expressed as mean ± SD; ***P<0.001 versus the control group.

Figure S2

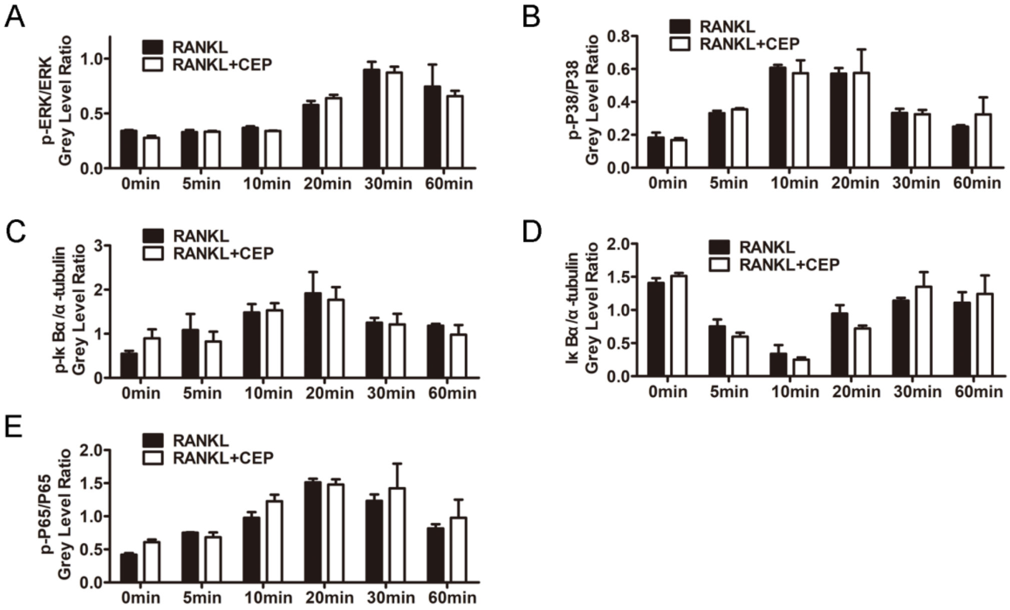

CEP did not suppress RANKL-induced activation of the ERK, P38 and NF-κB pathways in vitro. (A-E) The grey levels of phospho-ERK, phospho-P38 and phospho-P65 were analyzed by being normalized to total ERK, P38 and P65. The grey levels of phospho-IκBα and IκBα were normalized to α-tubulin. Values are expressed as mean ± SD; P<0.05 versus the control group.

Figure S3

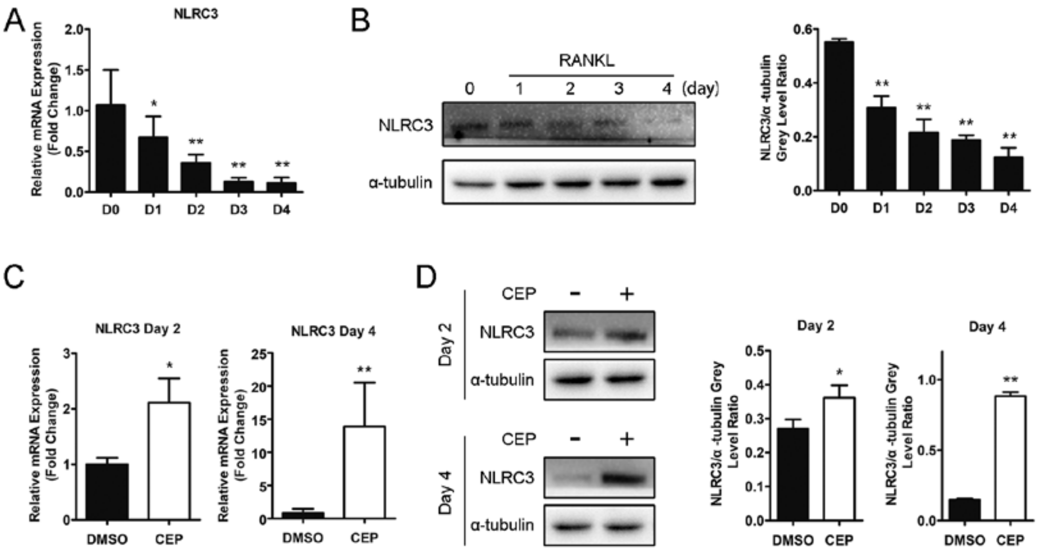

CEP inhibited the reduced expression of NLRC3 during osteoclastogenesis. (A) BMMs were cultured in osteoclastogenic medium for 0, 1, 2, 3 and 4 days. Total mRNA of samples from day0 to day4 were collected and analyzed by qPCR. (B) Total protein of samples from day0 to day4 were collected and analyzed by Western blotting using specific antibodies against NLRC3. The grey levels of NLRC3 analyzed by normalization to α-tubulin. (C) BMMs were cultured in osteoclastogenic medium for 1 or 3 days, followed by DMSO or CEP treatment for another 1 day. The mRNA expression levels of NLRC3 were analyzed by qPCR. (D) The protein expression of NLRC3 were detected by Western blotting. The grey levels of NLRC3 analyzed by normalization to α-tubulin. Values are expressed as mean ± SD; *P<0.05, **P<0.01 versus the control group.
